# Bioactivities and Microbial Quality of *Lycium* Fruits (Goji) Extracts Derived by Various Solvents and Green Extraction Methods

**DOI:** 10.3390/molecules27227856

**Published:** 2022-11-14

**Authors:** Katarzyna Rajkowska, Dorota Simińska, Alina Kunicka-Styczyńska

**Affiliations:** 1 Institute of Fermentation Technology and Microbiology, Faculty of Biotechnology and Food Sciences, Lodz University of Technology, Wólczańska 171/173, 90-530 Łódź, Poland; 2 Department of Sugar Industry and Food Safety Management, Faculty of Biotechnology and Food Sciences, Lodz University of Technology, Wólczańska 171/173, 90-530 Łódź, Poland

**Keywords:** goji, bioactivity, carotenoids, antioxidant activity, microbial safety, green extraction

## Abstract

Goji berries, known for their health-promoting properties, are increasingly consumed around the world, often in the form of naturally- or freeze-dried fruits, further unprocessed. The aim of the study was to obtain dried goji berries extracts, characterized by high bioactivity and safety in terms of microbial contamination for the consumer. In the study, various solvents (water, ethanol, acetone, ethyl lactate, sunflower oil) and green extraction methods (heating and ultrasound-assisted extraction (UAE)) were used. In goji extracts, antioxidant activity and carotenoids content as bioactivity indicators, as well as total number of microorganisms were determined. Boiling of powdered dried goji fruits in water resulted in extracts with the best features, i.e., with high antioxidant properties (2.75–4.06 μmol of Trolox equivalent (TE)/mL), moderate to high content of carotenoids (0.67–1.86 mg/mL), and a reduced number of microorganisms compared with fruits. Extracts in 50% ethanol and 50% acetone were characterized primarily by very high antioxidant activity (3.09–4.90 μmol TE/mL). The high content of carotenoids (0.98–3.79 mg/mL) and high microbial quality (<10 CFU/g) were obtained by extraction in ethyl lactate by the UAE method. The results could be useful in the development of functional food based on goji berry ingredients.

## 1. Introduction

Goji, also called goji berries or wolfberries, are the fruit of two closely related species *Lycium chinense* Mill and *L. barbarum* L. [1]. Both plant species are native to Asia, primarily to the northwest region of China known as the Ningxia Hui Autonomous Region. Commercial cultivation of wolfberries is also carried out in other regions of China (Xinjiang, Shaanxi, Gansu, Hebei), as well as in Mongolia, Japan, Korea, and Taiwan. In the first decade of the twenty-first century, goji berry cultivation began on a commercial scale in Canada, USA, and Europe [2]. In 2020, the goji market was valued at US $1.3 billion globally and is expected to grow by US $1.7 billion over the next 5 years [3].

Goji berries have been consumed as food and used in traditional Chinese medicine for over 4000 years [2]. Nowadays, goji fruits are usually consumed fresh, frozen, dried, or cooked and are also used in herbal teas, wines, and juices. Raw fruits deteriorate quickly after harvesting due to chemical alterations and microbial spoilage. Therefore, drying becomes one of the most important methods of goji berries processing to extend their shelf life [4]. For this reason, dried berries are the most popular and consumed goji products all over the world.

Goji is classified as a superfood due to its health-promoting functions in the human organism [5]. It may lower blood glucose and lipid levels, strengthen the immune system, regulate hormones, slow down the aging processes, prevent cancer, protect vision, and increase mental efficiency [4,5]. These beneficial effects are related to the high biological activity components in goji berries, mainly polysaccharides, carotenoids, and phenolics. Water-soluble polysaccharides, composed of pectic polysaccharides, glucan, xylan, and arabinogalactan proteins, are considered to be the most important bioactive constituents of goji berries [5,6]. The second highly significant group of biologically active compounds in goji fruits is that of carotenoids due to their beneficial effects on vision, retinopathy, and macular degeneration [1]. Goji berries are also a good source of phenolic compounds, including phenolic acids, flavonoids, phenylpropanoids, coumarins, lignans, and their derivatives [6].

The predominant carotenoid in goji is zeaxanthin in the form of zeaxanthin dipalmitate, reaching about 31–56% of the total carotenoids in goji berries [7]. Neoxanthin, cryptoxanthin, and β-carotene are also detected in goji extracts, but at much lower concentrations [8]. Zeaxanthin accumulates mainly in the retina and protects against age-related macular degeneration and cataracts [9]. Moreover, zeaxanthin has been shown to potentially protect against cardiovascular diseases, such as coronary heart disease and stroke. It should be emphasized that goji berries are the best natural source of zeaxanthin dipalmitate known so far [5].

Among the three main groups of compounds, the content of polysaccharides and phenolics strongly correlates with antioxidant properties of goji berries [1,5]. On the other hand, no significant correlation between antioxidant activities in vitro and total carotenoid content has been demonstrated [10], possibly due to the presence of zeaxanthin esters rather than free zeaxanthin in goji berries [6]. The content of bioactive substances is considered to be a good quality indicator of plants of pharmacological importance [11]. Since the antioxidant properties in vitro depend on the content of polysaccharides and phenols, we assume that the assessment of the antioxidant capacities and the level of carotenoids will allow for a proper and exhaustive determination of goji quality. 

Although goji has long history of consumption in China, it is not regulated in EU and US foods legislation regarding specific microbial limits and further food applications. According to the Codex Alimentarius (CAC/RCP 3-1969) [12], dried fruit should not contain any pathogenic microorganisms or any toxic substance of microbiological origin. Our previous research indicates the possibility of a high level of microbial contamination of dried goji berries (even above 10^6^ CFU/g) and of the presence of pathogenic microorganisms (unpublished data). In this context, the consumption of dried and further unprocessed goji berries may pose a health risk to the consumer.

Therefore, the aim of the study was to obtain goji extracts with high bioactivity, expressed as carotenoids content and antioxidant activity, as well as safe for the consumer. An additional advantage was the use of green solvents and green extraction methods in the study. Recently, numerous studies have focused on health benefits of goji berries, their antioxidant properties, and the content of phenolic compounds, whereas, to the best of our knowledge, there are no reports addressing both the bioactivity and microbial quality of goji.

## 2. Results and Discussion

Dried wolfberries are traditionally cooked before consumption, used as herbal tea, in soups, or as an addition to meat and vegetarian meals. For medical purposes, alcohol tinctures are also prepared from goji berries [1]. In this study, 12 various conditions for extraction of goji bioactive compounds were applied. With regard to the methods used in traditional Chinese medicine and cooking, dried goji berries were boiled in water at 100 °C for 30 min (W100/WB) or extracted in ethanol at a concentration of 25% (Et25/UAE), 50% (Et50/UAE), and 75% (Et75/UAE). In the study besides heating, in order to enhance extraction yield, ultrasound assisted extraction (UAE) was also applied as a clean and green extraction technology [13].

Moreover, in light of health and safety risks posed by many organic extraction solvents, in this study, ethyl lactate (EL100/UAE, EL100/WB) and sunflower oil (SO100/UAE, SO100/WB) were also applied as green and safe solvents. Both agents are considered to be low-toxic, efficient, biodegradable, and inexpensive [14,15]. Ethyl lactate has been approved by the U.S. Food and Drug Administration and European Food Safety Authority as a pharmaceutical ingredient and food additive [16]. Moreover, the compounds have previously been applied successfully to extract various phytonutrients, such as carotenoids, polyphenols, caffeine, and curcuminoids [14,15,16,17,18].

Finally, acetone at a concentration of 25% (Ace25/UAE), 50% (Ace50/UAE) and 75% (Ace75/UAE) was also used in the study, in accordance with Directive 2009/32/EC, which allows its use as extraction solvent during the processing of raw materials, foodstuffs, food components or food ingredients [19]. However, its use may only result in the presence of residues in technically unavoidable quantities, presenting no danger to human health.

### 2.1. Bioactivity of Goji Extracts

In the study, both the antioxidant activity of goji extracts and the content of carotenoids were determined as indicators of their bioactivity. The highest antioxidant capabilities were shown by extracts obtained in ethanol at a concentration of 25% and 50% (3.78–5.00 and 3.62–4.27 μmol TE/mL, respectively), and acetone also at a concentration of 25% and 50% (3.69–4.78 and 3.09–4.90 μmol TE/mL). The exception was extract of freeze-dried goji from Ningxia (G4) in 25% acetone, which antioxidant activity was significantly lower than that in 50% acetone and amounted to 2.52 μmol TE/mL (Table 1).

No antioxidant activity was found for any of the examined goji berries after extraction in ethyl lactate and sunflower oil (Table 1). In the other extracts, the lowest activity was determined in 75% acetone, ranging from 1.18 to 2.55 μmol TE/mL. Water extracts, regardless of the extraction method, were characterized by relatively high antioxidant properties, in the range of 2.31 to 4.31 μmol TE/mL in UAE method and 2.75 to 4.06 μmol TE/mL after boiling.

The content of carotenoids in goji extracts was highly diversified and depended on the extraction method, the solvent, and the type of fruit. The lowest concentrations of total carotenoids were obtained in extracts from natural dried goji berries from an unspecified region in China (0.33–1.52 mg/mL depending on the extraction conditions), and the highest in extracts from freeze-dried goji berries from Poland (0.51–4.56 mg/mL) (Table 1).

Generally, the highest concentrations of carotenoids were found in water extracts obtained by UAE method (freeze-dried goji from Poland G2 and natural dried goji from Ningxia G3) or in ethyl lactate (freeze-dried goji from Poland G2 and from Ningxia G4). The lowest content of carotenoids demonstrated extracts in sunflower oil (natural dried goji from China and from Ningxia), as well as ethanolic and acetone ones (freeze-dried goji from Poland and from Ningxia). It seems that the efficiency of carotenoid extraction was correlated with the method of fruit drying. Extraction in water was more effective method for naturally dried goji berries, whereas in ethyl lactate and sunflower oil for freeze-dried ones.

In this study, ethyl lactate and sunflower oil were selected as solvents not only because of their reduced risks to human health and protecting the environment, but also high efficiency of various phytonutrients extraction [14,15]. For both compounds as green equivalents of organic solvents, high usefulness in the extraction of both hydrophilic and lipophilic bioactive ingredients has been demonstrated [14,20]. The results presented here do not confirm neither the universality nor the high extraction yield of bioactive ingredients from goji berries. On the other hand, these results are consistent with previous reports suggesting that there is no significant correlation between total carotenoid content and antioxidant activities in in vitro studies [6].

The use of ultrasound was to enhance extraction yield and extend the range of solvent choice by replacing toxic organic solvents [15]. The cavitation force of ultrasound provides a greater solvent penetration into cellular materials, disrupts plant cell walls, and facilitates the release of intracellular components [13]. In recent years, numerous bioactive compounds have been extracted by UAE from several matrices, especially from fruits and vegetables [13,15,21]. In the present study, there was no significant increase in the extraction efficiency of compounds from dried goji berries by the UAE method compared with heating. In total, for 48 data that could be compared (antioxidant activity and total carotenoid content), no difference was found for 41.7% of the data, while for 33.3% better results were obtained for heating extraction than for ultrasound-assisted extraction.

### 2.2. Microbial Quality of Goji Extracts

**Figure 1:**
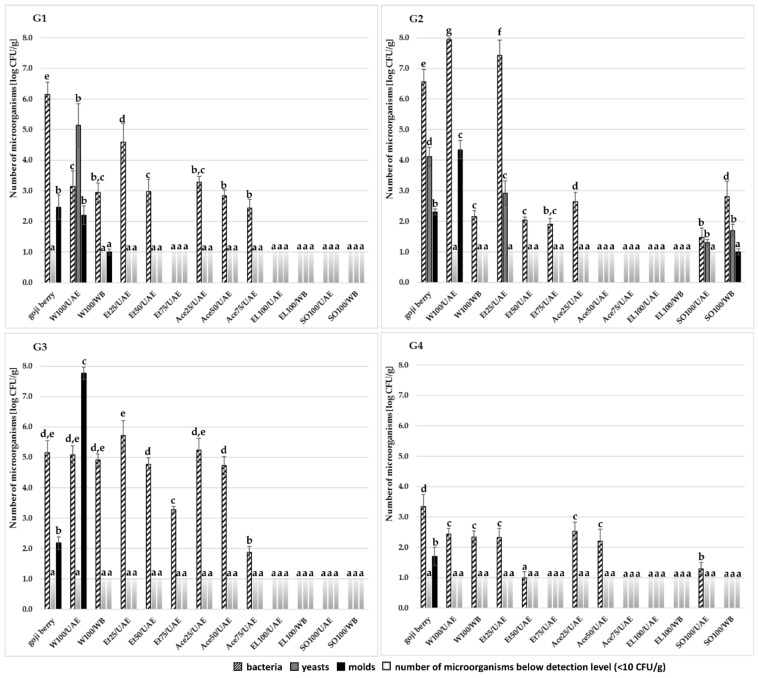
Total number of microorganisms in goji berries and extracts: G1—natural dried goji berries from an unspecified region in China; G2—freeze-dried goji berries from Poland; G3—natural dried goji berries from Ningxia, China; G4—freeze-dried goji berries from Ningxia, China. Means for each group of microorganisms within one goji type followed by the same letter are not significantly different (Tukey’s test, *p* < 0.05).

The level of microbial contamination of goji extracts was compared to the number of microorganisms in four tested dried goji berries of various origins. The water extracts obtained by the UAE method showed the lowest microbial quality, although in extracts of natural dried goji from China (G1) and freeze-dried berries from Ningxia (G4) a statistically significant reduction in the number of bacteria was observed compared with fruits (Figure 1). Similarly, a significant decrease in yeast counts was found in water extract of freeze-dried goji from Poland (G2), as well as in mold counts in extract of freeze-dried berries from Ningxia (G4). On the other hand, the number of yeasts in water extract (W100/UAE) significantly increased compared with natural dried fruits from China (G1), as well as the number of molds in extracts of freeze-dried berries from Poland (G2), and natural dried goji from Ningxia (G3).

The increase in the number of microorganisms may result from their rehydration during mild extraction conditions, i.e., water environment and temperature 40 °C for 30 min. According to FAO/WHO [22], dried fruits belong to the category of low-moisture foods, i.e., food products with low water activity, which contributes to an extended stability and prevents growth of many microorganisms. Despite the fact that microorganisms cannot reproduce in these products, they may persist for a long time [23]. The increase in the water activity in medium will then stimulate regeneration and activity of cells of microorganisms [24]. Moreover, low-frequency ultrasound, as in the UAE method in the study, can change the living state of cells, leading to increased proliferation and metabolism [25]. Furthermore, it has been hypothesized that ultrasound increases the rate of transport of oxygen and nutrients to cells and the rate of transport of waste products from cells, thus accelerating their growth [26].

More advantageous results in terms of microbial quality were obtained for water extracts boiled at 100 °C for 30 min (Figure 1). For all the tested fruits, a reduction in the number of microorganisms was found in these extracts, even by 4.5 log of bacteria, 3.1 log of yeasts, and 1.5 log of molds. Only for natural dried goji from Ningxia, no significant decrease in bacteria counts in water extract after boiling was observed, but the total number of microorganisms was more than 2 log lower than in fruits.

Interestingly, extracts in 25% ethanol of freeze-dried goji berries from Poland (G2) and natural dried goji berries from Ningxia (G3) showed a slight increase in the bacteria number by 0.8 and 0.6 log, respectively, but a significant reduction in the amount of molds to <10 CFU/g (Figure 1). Ethanol is known to be toxic to bacterial cells, but a bactericidal effect can be expected at concentrations between 60% and 85% [27]. Moreover, many bacteria and fungi are capable to grow even at high concentrations of organic solvents, and the response at the cellular level can be of a different nature. Membranes of cells cultured in the presence of alcohols may be more rigid due to a decrease in the lipid to protein ratio, but the increase of membrane fluidity has also been reported [28]. In light of these findings and the results obtained, it seems that 25% concentration of ethanol may be too low to effectively limit the number of bacteria in the extracts.

Goji extracts obtained in ethanol at a concentration of 50% and 75%, acetone at all the tested concentrations, ethyl lactate, and sunflower oil were characterized by high microbial quality and a significantly reduced amount of microorganisms in comparison with dried goji berries (Figure 1). The total number of microorganisms in these extracts ranged from <10 CFU/g to 6.0 × 10^4^ CFU/g, depending on the type of fruit and extract. However, the presence of pathogenic strains in these extracts cannot be unambiguously excluded, even at a very low level of microbiological contamination.

Recently, it has been reported that in low-moisture foods *Salmonella* spp., *Listeria monocytogenes*, Shiga-toxigenic *Escherichia coli*, *Bacillus cereus*, *Clostridium perfringens*, and *Cronobacter sakazakii* have been detected [23]. Pathogenic bacteria can remain viable in this type of food for at least months and cause food-borne diseases when consumed, posing a serious food safety risk [29].

### 2.3. Optimal Extraction Conditions

**Figure 2:**
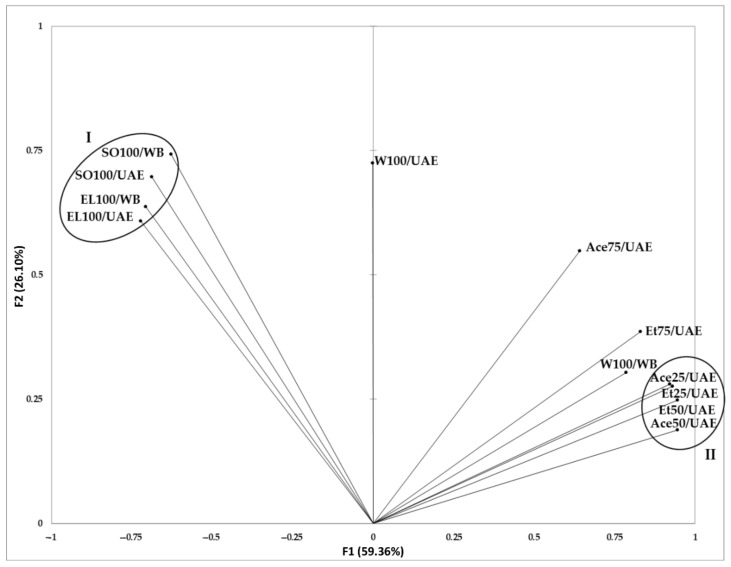
Principal component analysis (PCA) model of extraction methods used in the study, on the basis of bioactivity and microbial quality of the obtained extracts.

In order to compare the influence of the extraction conditions on the bioactivity and microbial purity of the goji extracts, a principal component analysis (PCA) was performed. In the PCA model, the extraction methods were divided into two main clusters (Figure 2). Cluster I contained goji extracts obtained in ethyl lactate and sunflower oil, which were characterized by lack of antioxidant activity, varied content of carotenoids, and high microbial quality. In cluster II, the extracts in ethanol and acetone at concentrations of 25% and 50% were grouped, all with the highest antioxidant capacity.

In the PCA model, water extracts obtained with both methods, as well as extracts in 75% ethanol and acetone were separated. Water extracts presented relatively high antioxidant activity and carotenoids content. However, the extracts received in water by boiling showed an increase in the number of microorganisms in comparison with fruits, and those obtained with the UAE method demonstrated only slightly better microbial quality. In contrast, the extracts in both 75% ethanol and 75% acetone exhibited very low contamination with microorganisms and moderate carotenoid content. Moreover, extracts in 75% acetone were also characterized by a very low antioxidant activity.

Summing up, the best properties, i.e., high antioxidant activity and moderate to high carotenoid content as well as high microbiological quality, were characteristic for water extracts obtained after boiling. The use of 50% ethanol or 50% acetone and UAE method allowed obtaining extracts with high antioxidant activity and microbiological safe for the consumer. A combination of ethyl lactate and ultrasound or heating appears to be the optimum treatment for efficient carotenoids extraction from goji berries and reduction of microbial contamination.

Previously, high usefulness of ultrasound-enhanced subcritical water extraction of goji berries (160 W, 110 °C, 5 MPa) in the extraction of polysaccharides has been demonstrated [30]. In addition to a higher yield of polysaccharides, their higher antioxidant and immunomodulatory activities have been found than in extracts obtained in boiling water, by ultrasonic extraction in water (360 W), or subcritical water extraction (110 °C, 5 MPa). Skenderidis et al. [31] developed optimal conditions of ultrasound-assisted extraction with regard to yield of the process, using water to raw material ratio of 30 mL/g, temperature 32.02 °C, ultrasonic power 30.95 W/cm^2^, and extraction time 32.03 min. As in our study, Povolo et al. [32] received results indicating the necessity to select a method for the expected results. They detected higher phenol concentrations in the Soxhlet extracts of dry goji fruits in ethyl acetate, whereas extracts in 80% methanol proved to have the highest antioxidant capacity. In accordance with our and other previously published data, type and concentration of the solvent used have also been shown to influence the bioactivity of goji berry extracts in terms of antioxidant activity and total polyphenol content [33].

## 3. Materials and Methods

### 3.1. Goji Berries

In this study, four *Lycium* sp. fruits of different origin were used, i.e., natural dried goji berries from an unspecified region in China (G1), freeze-dried goji berries cultivated in Poland (G2), natural dried goji berries from the Ningxia region in China (G3), and freeze-dried goji berries from the Ningxia region in China (G4). Goji berries named as G1 and G2 were commercially available and distributed by Radziowi Sp. z o.o. (Częstochowa, Poland) and Coactum Sp. z o.o. (Cracow, Poland), respectively. Sun-dried and freeze-dried goji berries from the Ningxia region were obtained courtesy of NingXia Senmiao Technology and Development Co., Ltd. (Chengdu, China).

### 3.2. Goji Berry Extracts

The goji fruits previously ground into powder were suspended in different solvents in the proportion 1:10 w/v. Extraction was carried out by heating in a water bath (Precisterm, JP Selecta) or in a ultrasonication bath (Sonorex Digitec, Bandelin). The solvents and extraction conditions are summarized in Table 2. After extraction, the samples were centrifuged at 4500 rpm for 15 min, and supernatants were collected.

### 3.3. Antioxidant Activity

The antioxidant capacity was determined using the Trolox-equivalent antioxidant capacity (TEAC) assay [34]. ABTS free radicals (ABTS^•+^) were obtained by reacting 7 mM aqueous solution of ABTS (2,2′-azino-bis(3-ethylbenzothiazoline-6-sulfonate); Roche, Mannheim, Germany) with 2.45 mM potassium persulfate (Sigma-Aldrich, Saint Louis, USA). This solution was stored in the dark at room temperature for 16 h in order to obtain free radicals, and then diluted so that the absorbance at 734 nm was 0.7 ± 0.02. Subsequently, 5 μL of the tested extracts was added to 1 mL of ABTS^•+^ solution, and after 3 min the absorbance was measured at 734 nm. The antioxidant activity associated with the neutralization of ABTS free radicals was expressed as μmol of Trolox equivalents per 1 mL of goji extracts. For this purpose, standard curves were prepared for Trolox (Merck, Darmstadt, Germany) solutions at a concentration of 0–10 μmol/mL in the appropriate solvents.

### 3.4. Total Carotenoid Content

The content of carotenoids in the extracts was determined by measuring absorbance at 450 and 503 nm against appropriate solvents as blanks. The concentration was calculated according to the following formula [35]:

C = 4.624 × A_450_ − 3.091 × A_503_ (1)

The total carotenoid content in extracts was expressed in mg/mL.

### 3.5. Determination of the Total Number of Microorganisms in Goji Extracts

In all goji berry extracts, the number of bacteria, yeasts, and molds was determined by the pour-plate method. In the assay, plate count agar (enzymatic digest of casein 0.5%, yeast extract 0.25%, glucose 0.1%, agar 1.5%; pH 7.0) was used for bacteria enumeration, dichloran glycerol agar (dextrose 1.0%, dichloran 0.0002%, magnesium sulfate 0.05%, monopotassium phosphate 0.1%, pepton 0.5%, agar 1.5%, glycerol 18%; pH 5.6) for yeasts, and malt extract agar (malt extract 2.0%, glucose 2.0%, peptone 0.1%, agar 2.0%; pH 5.4) for molds. The plates were incubated for 24–48 h at 30 °C (bacteria), 48–72 h at 28 °C (yeasts), and up to 5 days at 28 °C (molds).

For comparison, the number of microorganisms was also determined in goji fruits. For this purpose, 10 g of goji berries were weighed, transferred to 90 mL of 0.1% peptone water solution, and homogenized for 2 min. Enumeration of microorganisms was performed by the pour-plate method using microbial media and incubation conditions as described above.

### 3.6. Statistical Analysis

The results were collected in three independent experiments and expressed as arithmetic mean ± standard deviation. The significance of differences between means was determined using analysis of variance (one-way ANOVA) and Tukey HSD test, with *p* ≤ 0.05. A principal component analysis (PCA) model was generated based on the data of microbial quality of the extracts, their antioxidant activity, and carotenoids content to compare and group extraction methods in a statistically significant manner (XLSTAT 2022, Addinsoft, Paris, France).

## 4. Conclusions

The results of this study do not indicate the superiority of a particular extraction method, and the extraction conditions should rather be selected in accordance with the expected purpose. Thus, the results may be useful in the improvement of extraction targeted at specific compounds and could be of interest in the aspect of goji fruit processing during developing new goji-based supplements or food products. An important issue in the aspect of consumer safety is the microbiological quality of goji berries. The obtained results indicate the possibility of reducing microbial contamination in extracts, and also of obtaining extracts that are both microbiologically safe and characterized by high bioactivity. Further research directions should include the optimization of goji cultivation and processing methods in order to obtain healthy and safe food products with high bioactivity.

## Figures and Tables

**Table 1:** Antioxidant capacities and carotenoids content in goji berry extracts: G1—natural dried goji berries from an unspecified region in China; G2—freeze-dried goji berries from Poland; G3—natural dried goji berries from Ningxia, China; G4—freeze-dried goji berries from Ningxia, China.

Goji Berry Extracts		Antioxidant Capacities (μmol TE/mL) ^1^	Total Carotenoid Content (mg/mL)
G1	W100/UAE	4.31 ± 0.85 ^c,d^	1.41 ± 0.05 ^e^
	W100/WB	4.06 ± 0.21 ^c,d^	1.52 ± 0.28 ^e^
	Et25/UAE	5.00 ± 0.54 ^d^	1.51 ± 0.13 ^e^
	Et50/UAE	4.27 ± 0.62 ^c,d^	1.13 ± 0.17 ^b,c,d^
	Et75/UAE	3.52 ± 0.15 ^c^	1.05 ± 0.04 ^b,c,d^
	Ace25/UAE	4.78 ± 0.85 ^c,d^	1.25 ± 0.19 ^d,e^
	Ace50/UAE	4.90 ± 0.26 ^d^	1.19 ± 0.06 ^c,d,e^
	Ace75/UAE	2.55 ± 0.03 ^b^	0.92 ± 0.12 ^b^
	EL100/UAE	0 ^a^	0.98 ± 0.03 ^b,c^
	EL100/WB	0 ^a^	1.10 ± 0.01 ^b,c,d^
	SO100/UAE	0 ^a^	0.33 ± 0.05 ^a^
	SO100/WB	0 ^a^	0.36 ± 0.03 ^a^
G2	W100/UAE	2.54 ± 0.17 ^c^	4.56 ± 0.11 ^f^
	W100/WB	3.74 ± 0.18 ^e,f^	0.78 ± 0.04 ^a^
	Et25/UAE	4.09 ± 0.70 ^f^	0.63 ± 0.04 ^a^
	Et50/UAE	3.62 ± 0.28 ^d,e,f^	0.67 ± 0.05 ^a^
	Et75/UAE	2.78 ± 0.31 ^c,d^	1.30 ± 0.30 ^b^
	Ace25/UAE	3.69 ± 0.47 ^e,f^	0.59 ± 0.07 ^a^
	Ace50/UAE	3.09 ± 0.17 ^c,d,e^	0.51 ± 0.10 ^a^
	Ace75/UAE	1.18 ± 0.13 ^b^	1.30 ± 0.15 ^b^
	EL100/UAE	0 ^a^	3.79 ± 0.11 ^d^
	EL100/WB	0 ^a^	4.44 ± 0.12 ^e,f^
	SO100/UAE	0 ^a^	3.17 ± 0.27 ^c^
	SO100/WB	0 ^a^	4.05 ± 0.31 ^d,e^
G3	W100/UAE	2.99 ± 0.37 ^b,c,d^	2.12 ± 0.07 ^d^
	W100/WB	2.75 ± 0.35 ^b,c^	1.86 ± 0.13 ^c^
	Et25/UAE	3.78 ± 0.51 ^c,d^	1.92 ± 0.09 ^c,d^
	Et50/UAE	3.72 ± 0.41 ^c,d^	2.01 ± 0.06 ^c,d^
	Et75/UAE	3.17 ± 0.14 ^c,d^	1.89 ± 0.10 ^c^
	Ace25/UAE	3.73 ± 0.86 ^c,d^	1.93 ± 0.14 ^c,d^
	Ace50/UAE	3.98 ± 0.49 ^d^	1.87 ± 0.15 ^c^
	Ace75/UAE	1.90 ± 0.09 ^b^	1.14 ± 0.12 ^b^
	EL100/UAE	0 ^a^	1.97 ± 0.07 ^c,d^
	EL100/WB	0 ^a^	1.96 ± 0.06 ^c,d^
	SO100/UAE	0 ^a^	0.75 ± 0.11 ^a^
	SO100/WB	0 ^a^	0.72 ± 0.09 ^a^
G4	W100/UAE	2.31 ± 0.87 ^b,c^	0.74 ± 0.06 ^c^
	W100/WB	3.32 ± 0.12 ^c,d,e^	0.67 ± 0.07 ^b,c^
	Et25/UAE	4.09 ± 0.23 ^d,e^	0.66 ± 0.04 ^b,c^
	Et50/UAE	4.12 ± 0.85 ^e^	0.65 ± 0.04 ^b,c^
	Et75/UAE	2.84 ± 0.12 ^b,c,d^	0.62 ± 0.03 ^b,c^
	Ace25/UAE	2.52 ± 0.82 ^b,c^	0.55 ± 0.09 ^a,b^
	Ace50/UAE	4.03 ± 0.17 ^d,e^	0.60 ± 0.08 ^b,c^
	Ace75/UAE	1.83 ± 0.23 ^b^	0.41 ± 0.02 ^a^
	EL100/UAE	0 ^a^	1.53 ± 0.03 ^e^
	EL100/WB	0 ^a^	1.58 ± 0.04 ^e^
	SO100/UAE	0 ^a^	0.96 ± 0.19 ^d^
	SO100/WB	0 ^a^	0.97 ± 0.09 ^d^

^1^ TE—Trolox equivalents; data are expressed as mean ± SD of three independent experiments; means in a column for different extracts within one goji type followed by the same letter are not significantly different (Tukey’s test, *p* < 0.05).

**Table 2:** Conditions for the extraction of goji berries.

Designation	Solvent (Concentration)	Extraction Method	Conditions
W100/UAE	water (100%)	ultrasound assisted extraction	40 °C, 30 min, 35 kHz
W100/WB	water (100%)	heating in water bath	100 °C, 30 min
Et25/UAE	ethanol (25%)	ultrasound assisted extraction	40 °C, 30 min, 35 kHz
Et50/UAE	ethanol (50%)		
Et75/UAE	ethanol (75%)		
Ace25/UAE	acetone (25%)		
Ace50/UAE	acetone (50%)		
Ace75/UAE	acetone (75%)		
EL100/UAE	ethyl lactate (100%)		
EL100/WB	ethyl lactate (100%)	heating in water bath	45 °C, 60 min
SO100/UAE	sunflower oil (100%)	ultrasound assisted extraction	40 °C, 30 min, 35 kHz
SO100/WB	sunflower oil (100%)	heating in water bath	45 °C, 60 min

## Data Availability

All data generated or analyzed during this study are included in this published article, any questions may be addressed to the corresponding author.
